# Asymmetric introgression between fishes in the Red River basin of Texas is associated with variation in water quality

**DOI:** 10.1002/ece3.4901

**Published:** 2019-01-24

**Authors:** V. Alex Sotola, David S. Ruppel, Timothy H. Bonner, Chris C. Nice, Noland H. Martin

**Affiliations:** ^1^ Biology Department Texas State University San Marcos Texas

**Keywords:** fish, genetic structure, hybridization, introgression, water quality variation

## Abstract

When ecologically divergent taxa encounter one another, hybrid zones can form when reproductive isolation is incomplete. The location of such hybrid zones can be influenced by environmental variables, and an ecological context can provide unique insights into the mechanisms by which species diverge and are maintained. Two ecologically differentiated species of small benthic fishes, the endemic and imperiled prairie chub, *Macrhybopsis australis*, and the shoal chub, *Macrhybopsis hyostoma*, are locally sympatric within the upper Red River Basin of Texas. We integrated population genomic data and environmental data to investigate species divergence and the maintenance of species boundaries in these two species. We found evidence of advanced‐generation asymmetric hybridization and introgression, with shoal chub alleles introgressing more frequently into prairie chubs than the reciprocal. Using a Bayesian Genomic Cline framework, patterns of genomic introgression were revealed to be quite heterogeneous, yet shoal chub alleles were found to have likely selectively introgressed across species boundaries significantly more often than prairie chub alleles, potentially explaining some of the observed asymmetry in hybridization. These patterns were remarkably consistent across two sampled geographic regions of hybridization. Several environmental variables were found to significantly predict individual admixture, suggesting ecological isolation might maintain species boundaries.

## 1 INTRODUCTION

Speciation is usually not instantaneous (Grant, 1981; Levin, 1983, 2002; Wood et al., 2009), but rather involves the gradual buildup of reproductive isolation between diverging lineages (Coyne & Orr, 2004). Understanding the genetic and ecological processes involved in the evolution of reproductive isolation is an important goal for evolutionary biologists, and hybrid zones have been used as “windows” into the speciation process (Harrison, 1990). Hybridization occurs when divergent taxa meet and produce offspring of mixed ancestry and occurs in every major taxonomic group (Arnold, Sapir, & Martin, 2008). While hybridization can potentially be destructive to diversity at multiple scales, resulting in the erosion of genomic integrity, the fusion of taxa, or even the slow extirpation of taxa through genetic swamping (Allendorf, Leary, Spruell, & Wenburg, 2001; Edmands, 2007), hybridization can also act as an evolutionarily creative force leading to increased genetic diversity, adaptive introgression, and even hybrid speciation (Arnold & Martin, 2009; Arnold et al., 2008; Gompert & Buerkle, 2016; Martin, Bouck, & Arnold, 2005, 2006; Rieseberg et al., 2003; Soltis & Soltis, 2009). Whether hybridization is the result of species naturally coming into secondary contact, anthropogenically‐induced habitat modification, or introduction of closely related species (Rhymer & Simberloff, 1996), researchers have begun to recognize the importance of identifying the degree to which hybridization and introgression are occurring at both genomic and ecological scales to better understand evolutionary processes and inform conservation decision‐making.

The relatively recent ability to generate genome‐wide data for nonmodel organisms has fortunately been accompanied with appropriate computational tools to process these exceptionally large datasets (Buerkle & Lexer, 2008; Gompert & Buerkle, 2009, 2011, 2012; Mandeville, Parchman, McDonald, & Buerkle, 2015). This has enabled evolutionary biologists to ask questions about the nature of reproductive isolation and introgressive hybridization at a genomic scale (Mandeville et al., 2015; Sung, Bell, Nice, & Martin, 2018). Genome‐wide studies on nonmodel organisms have thus far provided strong support for the idea of a “genic view” of speciation (Wu, 2001), whereby the genomes of hybridizing species slowly accumulate loci that limit gene flow at very localized genomic scales and do not introgress, thus increasing reproductive isolation. However, introgression, either neutral or adaptive, can still occur throughout the remainder of the genome. Questions remain as to whether patterns of genomic isolation revealed in hybrid zones are consistent across divergent ecological contexts. The answers to these questions provide a context for predicting outcomes of hybridization and form the foundation for conservation management when hybridization involves species of concern.

In addition to an ecological effect, the direction of hybridization and introgression have been found to be influenced by the overall densities of the parental species simply because the rarer species have less opportunities to mate with conspecifics than with heterospecifics (i.e., the “Hubbs' effect”; Hubbs, 1955, Lepais et al., 2009). The causes of differences in densities have often been attributed to range shifts, mostly due to anthropogenic changes in the systems where these individuals occur (Perkin, Gido, Costigan, Daniels, & Johnson, 2015), although low amount of natural hybridization between sympatric species through natural range shifts have also been documented (Hasselman et al., 2014). Anthropogenic changes that can lead to hybridization, and a change in the local densities (or ranges) of species, include introductions of non‐native species (or translocation of native species to new watersheds), habitat fragmentation, habitat modification (Rhymer & Simberloff, 1996), or even purposeful fish stocking, resulting in a change in population densities (Heath, Bettles, & Roff, 2010; Lamaze, Sauvage, Marie, Garant, & Bernatchez, 2012; Marie et al., 2012). Additionally, changes in environmental variables (e.g. water quality), which can be altered via natural causes or anthropogenic disturbances, can increase the amount of hybridization between species (Marie et al., 2012; Yau & Taylor, 2013). Previous research has found an association between increased amounts of hybridization with a decrease in the available habitat. Further, hybridization in fish has been found to be associated with a number of environmental factors (e.g., dissolved oxygen, temperature, and pH) that are believed to be population limiting factors (Marie et al., 2012). Thus, understanding the ecological context in which hybridization is occurring is important in order to have a comprehensive understanding of the ecological drivers potentially influencing diversification and interspecific gene flow.

**Figure 1:**
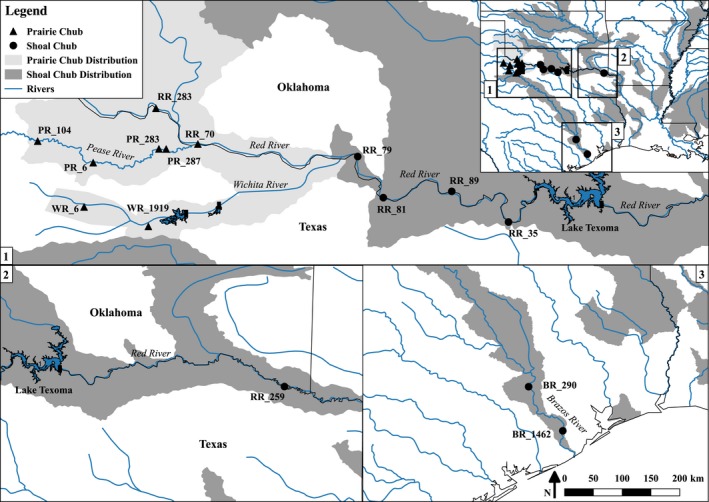
Map of locales where shoal chubs and prairie chubs were collected on the Red River (RR), Pease River (PR), Wichita River (WR), and Brazos River (BR). The map inset at the top right depicts the broad sampling frame statewide. Shapes denoting the sampling locations represent purportedly shoal chubs (circles) and prairie chubs (triangles) based on meristic morphological assignments. Light gray shading represents the prairie chub distribution, dark gray shading represents the shoal chub distribution (Data provided by NatureServe, 2010). Site codes are defined in Table 1

This study focused on two small benthic fishes, the shoal chub (*Macrhybopsis hyostoma*) and prairie chub (*Macrhybopsis australis*). These species are locally sympatric in the Red River along the border of Texas and Oklahoma upstream of Lake Texoma, an artificial reservoir created by the Denison Dam constructed in 1943 (Figure 1). The shoal chub has a broad distribution, occurring throughout the Mississippi River drainage and the West Gulf Slope drainages, including the lower reaches of Red River to upstream of the dam at Lake Texoma and the Brazos River (Echelle et al., 2018; Eisenhour, 2004). The prairie chub has a much more limited distribution and is endemic to the upper reaches of the Red River and several tributaries, including the Pease and Wichita rivers. The prairie chub currently is considered vulnerable and a species of greatest concern, whereas the shoal chub is a species of least concern (Jelks et al., 2008; Texas Parks & Wildlife Department, 2012). It was previously assumed that meaningful introgression does not occur between sympatric *Macrhybopsis* within the Red River, based on morphological analysis (Eisenhour, 2004). However, allozyme data revealed that shoal chubs of the Red River are more genetically similar to the endemic prairie chub than shoal chubs elsewhere (Echelle et al., 2018; Underwood et al., 2003). This suggests that either the prairie chub is not a distinct taxon worth conservation consideration but rather a morphologically distinct subpopulation of the shoal chub, or that these two genetically distinct species are hybridizing, and the geographic and genomic extent of such hybridization is unknown.

Here, we integrate population genomics and environmental data to investigate reproductive isolation and the maintenance of species boundaries between the shoal chub and prairie chub. Genotyping‐by‐sequencing (GBS) techniques were used to generate 39,122 SNPs which were then used to address three fundamental objectives: (a) quantify patterns of genetic structure and the geographic and genomic extent of hybridization, (b) examine patterns of excess ancestry at individual loci across two geographically disparate hybrid zones and identify the degree to which patterns of introgression were repeatable, and (c) determine the degree to which water quality parameters are associated with genetic structuring.

## 2 MATERIALS AND METHODS

### 2.1 Sample collection

A total of 15 sites were sampled for genetic analysis: two from the Wichita River, four from the Pease River, six from the Red River upstream from Lake Texoma, one downstream from Lake Texoma, and two from the Brazos River (Figure 1; Table 1). Seins were utilized to collect shoal chubs and prairie chubs from these sites. Specimens were euthanized with Tricaine Methanesulfonate (MS‐222, Western Chemical, Inc.), and then, subsequently stored in 95% ethanol.

**Table 1:** Collection locales of shoal chubs and prairie chubs with sampling code, river system, road crossing, latitude and longitude, sample size (*N*), and nucleotide diversity (π)

Code	River	Road crossing	Latitude	Longitude	*N*	π
BR_1462	Brazos River	1,462	29.34994	−95.58269	29	0.0038
BR_290	Brazos River	290	30.12944	−96.18693	30	0.0039
PR_104	Pease River	104	34.22786	−100.07375	8	0.0049
PR_283	Pease River	283	34.17915	−99.27841	7	0.0050
PR_287	Pease River	287	34.17982	−99.32343	19	0.0051
PR_6	Pease River	6	34.09471	−99.73016	27	0.0050
RR_259	Red River	259	33.68678	−94.69449	18	0.0053
RR_283	Red River	283	34.43119	−99.34181	17	0.0051
RR_35	Red River	35	33.72738	−97.15930	10	0.0057
RR_70	Red River	70	34.20985	−99.08233	10	0.0054
RR_79	Red River	79	34.13253	−98.09267	14	0.0057
RR_81	Red River	81	33.87807	−97.93435	14	0.0056
RR_89	Red River	89	33.91691	−97.51055	70	0.0055
WR_1919	Wichita River	1919	33.70029	−99.38871	66	0.0049
WR_6	Wichita River	6	33.82076	−99.78663	29	0.0049

### 2.2 DNA sequence generation, assembly, and variation

Genomic DNA was extracted from fin clips taken from a total of 384 individuals in 96‐well format using Qiagen DNeasy blood and tissue extraction kits and prepared for genotyping. For each sample, a reduced‐complexity genomic library was prepared for GBS protocols modified from (Gompert et al., 2012; Mandeville et al., 2015; Meyer & Kircher, 2010; Parchman et al., 2012). DNA from each individual was digested with the restriction enzymes EcoRI and MseI (New England Biolabs; NEB, Inc.). Fragments were labeled by ligating 8–10 base pair barcodes to the fragmented DNA. Two separate rounds of PCR were performed on these restriction‐ligation products using Illumina primers, and the final PCR products were pooled into a single library. This library was then sent to the University of Texas Genomic Sequencing and Analysis Facility (Austin, TX) and sequenced over two lanes on an Illumina HiSeq 4000 SR 150 platform after size selection between 300 and 400 base pairs via a Pippen Prep quantitative electrophoresis unit (Sage Science, Beverly, MA).

PhiX control sequences were identified by using Bowtie 2_db (Langmead & Salzberg, 2012). Raw reads that assembled to the PhiX genome were subsequently removed. A custom Perl script was used to remove Mse1 adapters and barcodes from sequence reads, correct single‐base sequencing mutations in barcodes, and match sample IDs with unique barcode identifiers. Because a reference genome is not available for *Macrhybopsis*, a de novo assembly was performed using part of the dDocent variant calling pipeline (Puritz, Hollenbeck, & Gold, 2014). Specifically, unique reads were found for each individual, and reads with less than four copies and shared across less than four individuals were filtered out of the dataset. The resulting filtered reads were assembled using CD‐hit (Fu, Niu, Zhu, Wu, & Li, 2012; Li & Godzik, 2006) with a threshold of 80% similarity. The scaffolds from this de novo assembly formed the basis of a reference‐based assembly in which all sequence reads were assembled to the reference scaffolds using the *aln* and *samse* algorithms from BWA 0.7.5a‐r405 (Li & Durbin, 2009). SAMtools ver. 0.1.19 and BCFtools ver. 0.1.19 were used to identify variable sites (Single Nucleotide Polymorphisms—SNPs) and to calculate the Bayesian posterior probability that individual SNPs were variable (Li et al., 2009). In order for a locus to be included in the dataset, a minimum of 50% of all sampled fishes must have had at least one read at a particular locus (i.e., the “d” parameter in BCFtools was set at 0.5). For contigs containing more than one SNP, only a single randomly chosen SNP was used for subsequent analyses. Importantly, individual SNP genotypes were not “called,” but rather genotype likelihood estimates were assigned for each variable site for each individual. Furthermore, population allele frequencies were estimated directly from these genotype likelihood estimates, and SNPs with minor allele frequency of <0.05 were excluded from the dataset. In all, genotype likelihood data were obtained for a total of 39,122 SNPs and used for population genomic analyses in this study.

### 2.3 Genetic structuring and diversity

To examine the genetic structuring of the shoal and prairie chubs, population genetic parameters were estimated using Entropy (Gompert et al., 2014a, 2014b; Mandeville et al., 2015). Entropy is a hierarchical model whereby an individual's assignment probability to each of any number of preassigned populations is estimated in a Bayesian framework. While interpretation of the output is similar to that of Structure (Falush, Stephens, & Pritchard, 2003; Pritchard, Stephens, & Donnelly, 2000), Entropy accounts for variation in sequence coverage, sequence alignment, and genotyping errors, and produces posterior genotype probability distributions using prior probabilities from cluster allele frequencies (Gompert et al., 2014a). Models with different numbers of populations (*k* = 2–4) were compared; no attempts were made to identify the “best” *k*, but results of *k* = 2–4 runs are reported here, as an examination of all values of *k* all could provide a more comprehensive understanding of population structure. Posterior distributions of genotypes and admixture proportions were calculated for each *k* using Markov Chain Monte Carlo (MCMC) with 100,000 iterations sampling every 10th iteration. The first 5,000 iterations were discarded and each model for all *k* clusters was run twice. Calculation of the Gelman–Rubin diagnostic statistic and effective sample sizes were used to check chain convergence, and genotype and admixture proportions were subsequently averaged across both runs of each model. Posterior distributions for parameters were summarized as means, medians, and 95% credible intervals.

Population differentiation was explored by calculating pairwise Nei's *G*_ST_ (Nei, 1987). Allele frequencies were calculated in R (R Core Team, 2017) from the mean genotype posterior probabilities, which were in turn used to calculate pairwise *G*_ST_ values. In addition, population‐level variation for each locality was reported using the genetic diversity index (π) calculated with SAMTools using the expectation–maximization (EM) algorithm, employing 20 iterations for each collection locale to achieve convergence of estimates (Li, 2011). In order to summarize the distribution of genetic variation, principal component analysis (PCA) was performed in R on the genetic covariance matrix calculated from the genotype probability estimates generated in Entropy (Gompert et al., 2014a).

### 2.4 Genetic and environment associations

A bidirectional stepwise regression was run to determine if the location of each species and hybrid individuals could be predicted by one or more environmental variables, which are known drivers of fish communities and can cause mortalities if they reach above or below tolerance levels (Barlow, 1958; Ostrand, 2000; Ostrand & Wilde, 2001). Environmental data were collected during sampling events throughout the year as part of a larger project assessing the population dynamics and status of prairie chubs (Ruppel et al., 2017). Four environmental variables—specific conductance (µS/cm), pH, temperature (^o^C), and dissolved oxygen (mg/L) were measured with a YSI 556 multiprobe sonde, and two additional variables, depth (m), and current velocity (m/s), were measured with a Marsh‐McBirney Flo‐mate model 2000 electromagnetic flowmeter. For the Red River basin collections, these environmental variables were incorporated into a bidirectional stepwise multiple regression model to assess whether such variables could explain a significant portion of the variation in genetic assignment probabilities (*q* from *k* = 2) calculated from Entropy for the Red River basin fish only. Once terms deemed not useful by stepwise selection for use in the final model were removed, using the package *relaimpo*, relative importance (percent *R^2^* explained) of each environmental variable was assessed using the “lmg” type performing 10,000 bootstraps to determine confidence intervals for each variable's relative importance (Groemping, 2006). This analysis was done in R, and all data were log transformed prior to analysis.

### 2.5 Admixture class

While estimates of genome‐wide admixture proportions can indicate whether or not hybridization is ongoing between divergent taxa, such estimates do not provide evidence as to how such admixture is occurring. Identifying admixture classes can potentially provide a more detailed look at the long‐term stability of hybrid zones and help to determine the extent to which both current hybridization and long‐term introgression are occurring. For example, if most of the admixed individuals are found to be early‐generation hybrids, this can indicate that either hybridization is a relatively recent phenomenon, or that late‐generation hybrids may be largely unfit as they are not encountered. If, on the other hand, admixed individuals are shown to be of late‐generation hybrids, this can indicate that the hybrid zone has been long established and that introgression across species boundaries is a possibility. Thus, we used an admixture class model in Entropy to estimate admixture classes (Q_12_; Gompert et al., 2014a). This analysis assumes two source species; therefore, it was performed only on the Red River basin fishes due to the high degree of genetic differentiation between them and the Brazos River fish. We ran two independent MCMC analyses with 15,000 iterations, sampled every 5th iteration after a burn‐in of 5,000 iterations. Admixture classes were estimated from samples of both independent MCMC analyses.

### 2.6 Symmetry of introgression

The Bayesian genomic cline (BGC) model (Gompert & Buerkle, 2011, 2012) was used to quantify genome‐wide variation in introgression among admixed individuals in two geographically separate areas. Because significant associations between water quality parameters and genetic assignment were found, these areas were run separately in an attempt to discover whether or not patterns of allelic introgression differed among divergent environmental conditions. The first location included hybrid individuals identified in the lower reach, just upstream from Lake Texoma at sampling locations RR_89, RR_81, RR_35, and RR_79 (downstream reach, *N* = 78), while the second included hybrid individuals identified from locations RR_283, PR_287, PR_6, and WR_1919 (upstream reach, *N* = 16; see Results, Figure 1). Only individuals with Q_12_ (interpopulation ancestry) values above 0.05 were included in the separate analyses. BGC is a hierarchical model that examines the probability of ancestry (ranging from 0 to 1) at individual loci as a function of an individual's hybrid index (*h*; also ranging from 0 to 1). Two locus‐specific parameters were estimated, α and β. These reflect either an increase (+ α) or decrease (−α) in the probability of shoal chub ancestry for a locus relative to the probability of hybrid ancestry, while the parameter β specifies an increase (+ β) or decrease (−β) in the rate of change, with positive values indicating steeper clines and limited rates of introgression between species and negative values indicating wider clines with increased rates of introgression (Gompert & Buerkle, 2011; Gompert et al., 2012; Parchman et al., 2013). In order to estimate the marginal posterior probability distributions for α and β*,* two independent chains of MCMC were performed each with 50,000 iterations, sampled every 5th iteration, and following a 25,000 iteration burn‐in. Outputs of the two chains were combined after determining both converged to the same stationary distributions. Medians and 95% CIs are reported for α and β, exceptional loci were identified as those where the 95% CIs of the parameter value did not intersect zero.

The degree to which exceptional α loci identified in the upstream reaches were also identified as exceptional in the downstream reaches was assessed by calculating the probability (*p*) that these two sets of loci were associated simply by chance using the following formula (from Sung et al., 2018):

$$\sum_{p=m}^{s}p=\frac{\left[\begin{array}{c} l\\ m \end{array}\right]\times \left[\begin{array}{c} n-l\\ s-m \end{array}\right]}{\left[\begin{array}{c} n\\ s \end{array}\right]},$$

where *l* is the number of exceptional loci identified in the larger (downstream) hybrid zone, *s* is the number of exceptional loci identified in the smaller (upstream) hybrid zone, *m* is the number of exceptional loci shared across both hybrid zones, and *n* is the total number of SNPs in the sample (e.g., 39,122 in the current study). This was only calculated for the α parameter as no exceptional β loci were found in the upstream hybrid zone.

## 3 RESULTS

### 3.1 Field and genomic sampling

Sample sizes of fishes captured from the 15 sampling locales ranged from seven to 70 (Table 1). DNA sequencing resulted in a total of 409,970,747 reads with an average of 1,070,419 sequences per individual. Individuals with low coverage (mean of <2 reads per locus) were not included in analyses, resulting in a total of 368 individuals with an average of 7.01 (*SD* = 1.62) reads per locus per individual. Overall, a total of 39,122 SNPs were ultimately included in the analyses.

### 3.2 Genetic structuring and diversity

Genotype likelihood estimates were calculated for all SNPs for each individual. Highest π diversity levels were found in the Red River sites upstream from Lake Texoma, followed by the Wichita and Pease rivers, with the lowest observed in the Brazos River (Table 1). The highest amount of genetic differentiation (*G*_ST_) was found between the Brazos River sites and all the Red River drainage sites, indicating higher relative genetic differentiation between shoal chubs of different drainages than shoal chubs and prairie chubs within the Red River drainage. Next highest levels of differentiation were found with the site downstream from Lake Texoma (RR_259) compared to the Pease and Wichita rivers sites. Lowest relative genetic differentiation was found among the Pease and Wichita River sites and among the mainstem Red River sites (Table 2 and Appendix Table S1).

**Table 2:** Pairwise genetic differentiation (Nei's *G*_ST_) between all sites where genetic analyses were performed

	BR_290	PR_104	PR_6	PR_283	PR_287	RR_89	RR_81	RR_35	RR_79	RR_70	RR_283	RR_259	WR_1919	WR_6
BR_1462	0.004	0.391	0.383	0.376	0.383	0.229	0.192	0.229	0.293	0.317	0.387	0.189	0.383	0.386
BR_290		0.385	0.376	0.370	0.377	0.224	0.187	0.224	0.287	0.311	0.380	0.184	0.377	0.379
PR_104			0.015	0.017	0.016	0.075	0.110	0.097	0.034	0.024	0.025	0.166	0.013	0.015
PR_6				0.009	0.008	0.068	0.103	0.090	0.027	0.016	0.017	0.158	0.005	0.006
PR_283					0.010	0.064	0.098	0.085	0.026	0.017	0.018	0.152	0.007	0.009
PR_287						0.069	0.103	0.090	0.028	0.017	0.018	0.159	0.006	0.008
RR_89							0.010	0.010	0.022	0.047	0.073	0.029	0.068	0.070
RR_81								0.014	0.043	0.073	0.107	0.020	0.103	0.105
RR_35									0.036	0.065	0.094	0.029	0.089	0.092
RR_79										0.020	0.034	0.081	0.026	0.028
RR_70											0.025	0.121	0.015	0.017
RR_283												0.162	0.015	0.017
RR_259													0.159	0.162
WR_1919														0.005

**Figure 2:**
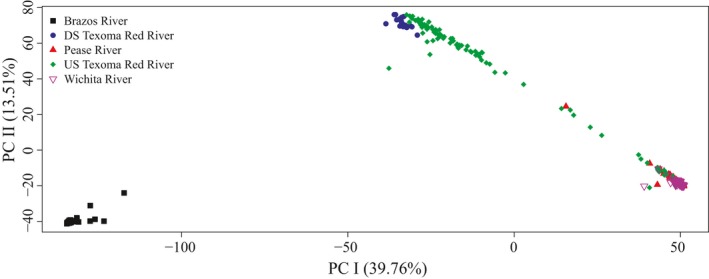
PCA of genetic differentiation of all individuals from each collection locale. PC I explains approximately 39.76% of the genetic variation in the data, and PC II explains approximately 13.51% of the variation. Shapes and colors represent different collection locales. Black squares are individuals from the Brazos River, blue circles are individuals captured downstream of Lake Texoma on the Red River, red triangles are individuals from the Pease River, green diamonds are individuals captured upstream of Lake Texoma from the Red River, and purple triangles are individuals captured from the Wichita River

Principal component I explained 39.76% of the variation, and principal component II explained 13.51% of the variation in the genotypic data (Figure 2). Three primary clusters were indicated by PCA: (a) shoal chubs from the Brazos River, (b) prairie chubs from the Pease, Wichita, and upper Red River sites, and (c) shoal chubs from the downstream Red River sites. PC I segregated shoal chubs from the Brazos River sites from shoal chubs and prairie chubs from the Red River drainage, while PC II segregated prairie chubs from the Pease and Wichita rivers sites from shoal chubs from the Red River and Brazos River sites. Hybridization between the shoal chub and prairie chub is evident from intermediate individuals observed from the mainstem Red River sites and Pease rivers sites with intermediate PC I and PC II scores (Figure 2).

**Figure 3:**
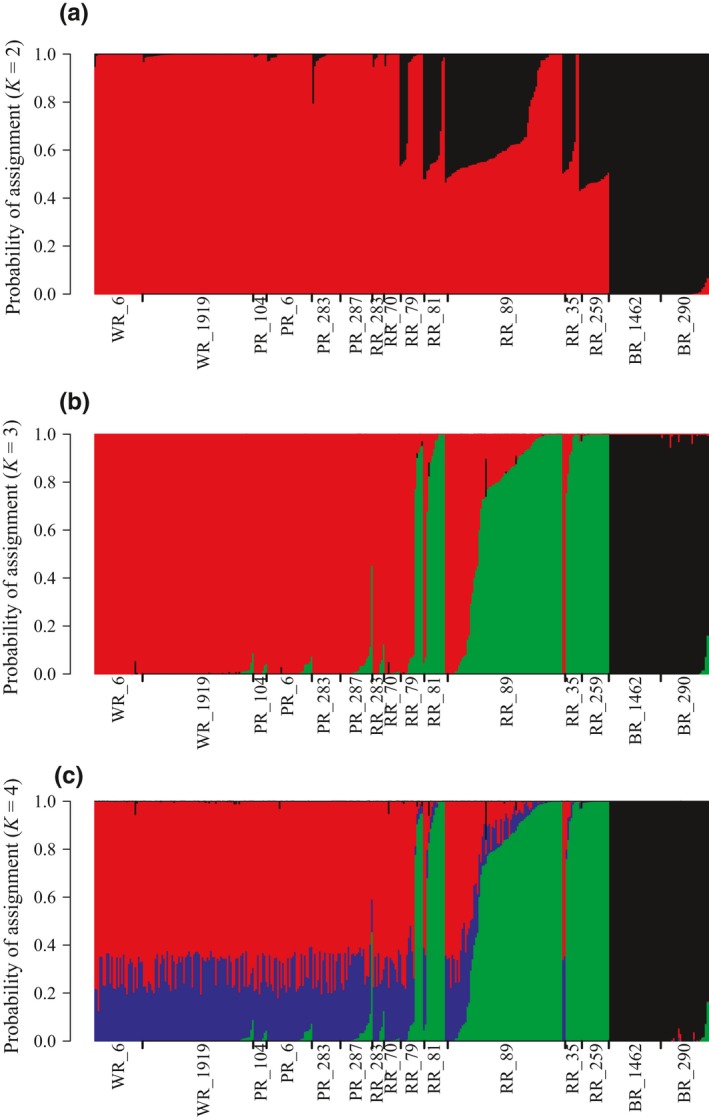
Entropy plots for *k* = 2 (a), 3 (b), and 4 (c) for all sampled sites oriented from most upstream to downstream (left to right) in the Red River basin, followed by the Brazos River. Site codes are defined in Table 1

Admixture proportions were calculated in Entropy for *k* = 2–4 with all of the sampled sites (Figure 3). Similar to the PCA results, for *k* = 2, the model separated individuals from the Brazos River and Red River drainages into two genetic clusters. Shoal chubs in the Red River show mixed ancestry between the two genetic clusters. At *k* = 3, individuals from the two Brazos River sites (Brazos River shoal chub) were separate from Red River sites. All the individuals sampled from the site downstream from Lake Texoma, RR_259, along with some individuals from sites upstream from Lake Texoma (RR_35, RR_89, RR_81, and RR_79) are grouped into the second (Red River shoal chub) cluster with very high probability. The third genetic cluster (prairie chubs) consisted of individuals from the Wichita, Pease, and upstream Red River sites (RR_283, RR_70). A majority of individuals from RR_79 downstream to the site just upstream of Lake Texoma (RR_35) had intermediate assignment probabilities between the Red River shoal chub and prairie chub genetic clusters, indicating a clear hybrid zone. For *k* = 4, genetic clusters resemble those observed in *k* = 3, although no individuals had 100% assignment probability to additional clusters that were added and do not appear to provide any easily discernable biological interpretations.

### 3.3 Genetic and environmental associations

Bidirectional stepwise selection for water quality variables predicting assignment probability from Entropy (*q*) found the model with the lowest AIC score included specific conductance, depth, dissolved oxygen, pH, and current velocity; temperature was removed by the stepwise procedure. The selected model was significant (*F*_5,303_ = 128.5, *p* < 0.001) and explained approximately 67.43% of the variation in *q* (Table 3). Of the explained variation in the final model, specific conductance (95% CI: 34.36 < 40.08 < 45.80) explained the highest percentage. Specific conductance, current velocity, and pH were found to have a positive relationship with *q*, indicating that as these variables increase, prairie chub ancestry also increases; the opposite was found for dissolved oxygen and depth.

**Table 3:** Output from multiple regression of environmental variables (specific conductance, water depth, dissolved oxygen, current velocity, and pH) predicting *q* from Entropy (assignment probability to first genetic cluster from Entropy), including estimated slopes, *t*‐values, *p*‐values, percent *R* ^2^ explained, and upper and lower 95% confidence intervals for percent *R* ^2^ explained

Term	Estimate	*T*‐value	*p*‐value	% *R* ^2^ Explained	Lower 95% CI	Upper 95% CI
Specific conductivity	0.538	8.793	<0.001	40.08	34.36	45.80
Depth	−0.287	−3.529	<0.001	34.83	31.04	38.16
Dissolved oxygen	−2.374	−7.023	<0.001	20.30	14.58	25.80
Current velocity	0.856	4.158	<0.001	3.87	2.76	5.62
pH	5.055	3.112	0.002	0.91	0.27	3.19

### 3.4 Admixture class

**Figure 4:**
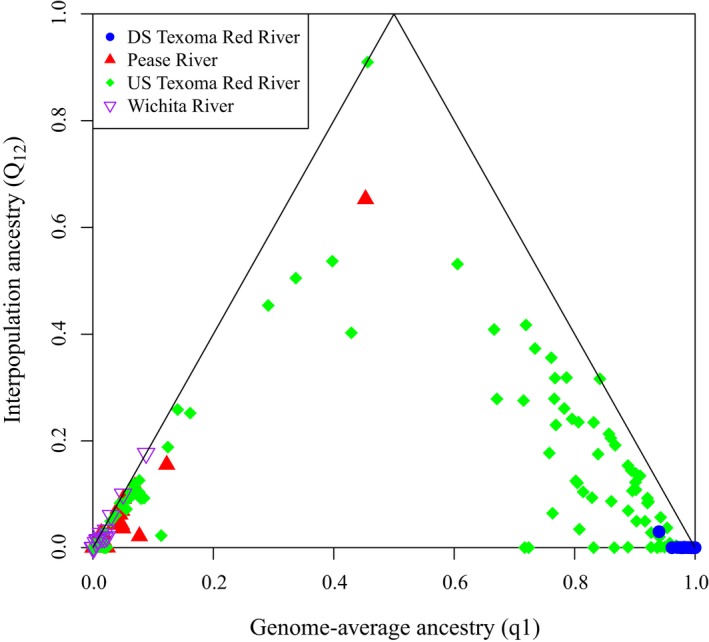
Scatter plot showing the relationship between genome‐average ancestry (q1) and interpopulation ancestry (*Q*_12_). Symbols correspond to individuals from areas of the Red River basin (DS Texoma Red River = RR_259; Pease River = PR_287, PR_283, PR_6, and PR_104; US Texoma Red River = RR_35, RR_89, RR_81, RR_79, RR_70 and RR_283; Wichita River = WR_1919 and WR_6). Solid lines indicate the maximum possible interpopulation ancestry given global genetic ancestry

Admixture class estimates (*Q*_12_) revealed mixed ancestry for individuals from several sites upstream from Lake Texoma (Figure 4). Individuals collected from these sites had genomic regions that were inherited from two different source species (nonzero *Q*_12_), indicative of hybridization. All of the individuals taken downstream from Lake Texoma (RR_259) were of pure shoal chub ancestry. A majority of individuals from the Pease River, upper Red River sites (RR_283 and RR_70), and the Wichita River were predominately of prairie chub ancestry, although a minority of individuals did have small amounts of mixed ancestry. No pure shoal chub was captured upstream of RR_81. A majority of the individuals from sites upstream of Lake Texoma (RR_35, RR_89, RR81, and RR_79) are not easily assignable to early‐generation hybrids due to backcrossing between hybrid lineages and parental species (Nadeau, 2014), yet one individual does appear to be genetically indistinguishable from an F_1_ hybrid (individual nearing 0.5 for genome‐average ancestry and 1.0 for interpopulation ancestry), indicating that while F_1_ hybridization may be rare, those hybrids—as well as later‐generation hybrids, are likely to be fit.

### 3.5 Genomic clines

**Figure 5:**
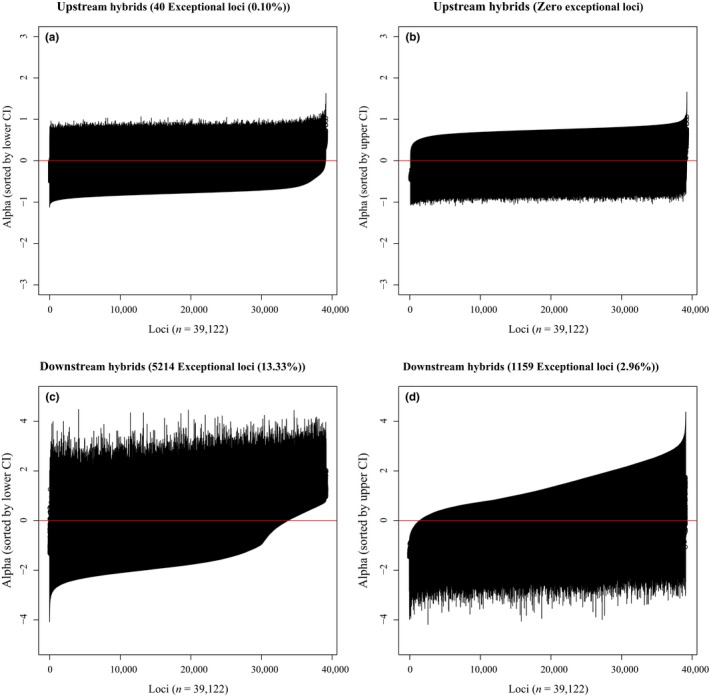
Median (± 95% CIs) of Bayesian genomic cline parameter α sorted by the lower 95% CI and by the upper 95% CI for hybrid individuals captured in the upstream (a, b) and downstream Red River (c, d). Upstream hybrids include individuals captured at RR_283, PR_287, PR_6, and WR_1919; downstream hybrids include individuals captured at RR_89, RR_81, RR_35, and RR_79. Number of loci considered exceptional due to their 95% CI not overlapping zero are given in the title of each respective plot

In hybrid individuals captured in the upstream reach, the posterior estimates of genomic cline parameter α was variable across loci, ranging from −0.47 to 1.05 (Figure 5). The parameter β was less variable with posterior estimates ranging from −0.15 to 0.06. In total, 40 loci (0.10%) were found to be exceptional (95% credible intervals do not include zero), all of which had positive α values, meaning that these shoal chub alleles were likely selectively favored regardless of the genomic background in which they occurred. None of the loci in this upper reach were found to have exceptional β values.

In hybrid individuals from the downstream reaches, α was again highly variable across loci, ranging from −1.43 to 2.03, while measures of β ranged from −1.08 to 0.81. In total, 6,393 loci (16.34%) were found to have exceptional α values; 5,214 of those had positive α values (i.e., shoal chub alleles had higher frequency regardless of the genomic background), while 1,159 had negative α values (i.e., prairie chub alleles had higher frequency regardless of the genomic background; Figure 5c,d), two loci revealed exceptionally positive β values (i.e., were overrepresented in conspecific backgrounds, while underrepresented in heterospecific backgrounds—consistent with loci affecting reproductive isolation and are likely associated with reproductive isolation between the two taxa), and 18 had exceptionally negative β values (indicating that bidirectional selective introgression is likely to occur for these loci). We found significant concordance (*p* < 0.0001) across the two hybrid zones. Of the 40 exceptional loci found in the individuals sampled in the upstream reach, 35 loci were also identified as exceptional loci in individuals sampled in the downstream reach (Figure 5a,b). This is consistent with those loci imparting a selective advantage throughout the range where hybrids occur, with such selective advantages not being site‐specific.

## 4 DISCUSSION

### 4.1 Genetic structuring and hybridization

Previous studies utilizing small genetic datasets and morphological data concluded that shoal chubs and prairie chubs were morphologically distinct species with no meaningful hybridization and introgression occurring (Echelle et al., 2018; Eisenhour, 2004; Underwood et al., 2003). The current genomic results corroborate that the two species are in fact distinct taxa, yet there is evidence of interspecific hybridization in the lower reaches of the river basin where they co‐occur. Hybridization occurs predominately in the reach immediately upstream from Lake Texoma, with much less hybridization in the upper Red River and Pease River and trace amounts of admixture evident in the Wichita River. As in previous studies, we found higher genetic differentiation between two populations of shoal chubs in different drainages (Brazos River and Red River) than between shoal chubs and prairie chubs within the Red River (Echelle et al., 2018; Underwood et al., 2003). We found that collection locales consisting predominately of hybrid individuals had higher genetic diversities (π), which is expected if alleles from divergent taxa are contributing to allelic diversity at these collection locales (Zalapa, Brunet, & Guries, 2010). Additionally, there is asymmetry in the hybrid zone, with hybrid individuals consisting predominately of shoal chub background, and introgression of alleles, with shoal chub alleles introgressing at a higher frequency than prairie chub alleles. Attempts to classify the hybrid individuals into early‐generation hybrid classes (e.g., F_1_, F_2_, or BC_1_ individuals) were largely unsuccessful, with only a single mixed‐ancestry individual being categorized as a possible F_1_. All other hybrid individuals were late‐generation hybrids that were not easily categorized into specific hybrid classes (Nadeau, 2014), likely indicating that hybridization has persisted for many generations. This is likely because in the hybrid zone, a majority of the sampled individuals were hybrids, with only a few pure individuals of each species, thus there is little potential for early‐generation hybridization to occur.

The hybrid zone has a broad pattern of asymmetry. Asymmetric hybridization been attributed to several factors including differences in generation time (Barton, 1986), mating behavior (Konkle & Philipp, 1992; Lamp & Avise, 1986), fitness (Ostberg, Slatton, & Rodriguez, 2004), or relative abundances of the parental species (Lepais et al., 2009). Relative abundances of parental species differ within the hybrid zone, with shoal chubs being much more common than prairie chubs. In the reach where hybridization occurs, relative abundances of putatively identified shoal chubs (range 2.18%–2.83%) are approximately 3 to 4 times higher than that of putatively identified prairie chubs (0.31%–0.86%; Ruppel et al., 2017). This potentially results in pure prairie chub and hybrid individuals having more mating opportunities with heterospecifics than with conspecifics. Greater abundance of shoal chubs and lesser abundance of prairie chubs within the hybrid zone corresponds with an overlap in distributions between prairie chubs and shoal chubs indicating this hybrid zone might be a natural secondary contact zone between the two species and therefore represent natural hybridization between closely related taxa. Lake Texoma, which is located downstream of the hybrid zone, might have exacerbated hybridization between these two species. Dams disrupt the habitat and environmental heterogeneity of rivers, homogenizing habitats (Santucci, Gephard, & Pescitelli, 2005), which can lead to an increase in introgressive hybridization (Hasselman et al., 2014; Seehausen, Takimoto, Roy, & Jokela, 2008). Thus, it is possible that the construction of Lake Texoma could have restricted a species of mobile *Macrhybopsis* (Wilde, 2016; Worthington et al., 2016) upstream and, along with altering upstream habitats (e.g., deeper water, more similar to habitats associated with shoal chubs; Eisenhour, 2004), could have anthropogenically inflated shoal chub numbers in the zone.

Bayesian genomic cline analyses demonstrated that introgression rates were quite variable across the genome. This comports with other studies examining genome‐wide rates of introgression in hybrid zones (Gompert et al., 2014a, 2014b, 2012; Kingston, Parchman, Gompert, Buerkle, & Braun, 2017; Parchman et al., 2013; Payseur, 2010; Teeter et al., 2010; Yuri, Jernigan, Brumfield, Bhagabati, & Braun, 2009) including fish (Nolte, Gompert, & Buerkle, 2009; Schaefer, Duvernell, & Campbell, 2016). Of the 39,122 loci examined in the current study, 16% were revealed to have exceptional α values in the downstream reach, with shoal chub alleles having a >5.5‐fold chance of introgressing across species boundaries. The same pattern was observed in hybrids in the upper reach; while only 40 loci with exceptional α values were found in that hybrid zone, all of them were revealed to be crossing from shoal chubs to prairie chubs. The fact that such a small number of loci were found to have exceptional α values in individuals captured in the upper reach is not surprising given that the number of hybrid individuals used in this analysis was quite small (*N* = 16). Exceptional α values are consistent with selection or adaptive introgression, thus, it is possible that these alleles are selectively advantageous and have spread into the alternative genomic background (Whitney, Randell, & Rieseber, 2006). This indicates that some of the asymmetric introgression observed here could be explained by the fact that shoal chub alleles are more often than not selectively advantageous. However, stochastic evolutionary processes (i.e., drift) in small populations can also contribute to increased exceptional α values. It is difficult to know if the populations of chubs in the Red River are small and potentially influenced by drift, yet similar levels of genetic diversities and the fact that relative abundances have largely increased or remained stable since the 1940s in the areas sampled by this study (Ruppel et al., 2017) suggest drift should not be acting strongly on these fishes. Thus, selection or adaptive introgression seem the likely drivers of the high amount of exceptional α values in these fishes, perhaps some of which is due to several extrinsic factors throughout the basin.

In this study, of the 40 exceptional loci found in hybrid individuals captured in the upstream reach, 35 of them were also found to be exceptional in the downstream reach, and this overlap was greater than expected by chance. This likely indicates that the selective advantages afforded by these loci are not simply site‐specific, but occur basin‐wide, and these loci are strong candidates for having moved upstream into largely pure prairie chub populations via selection, especially as no pure shoal chub individuals are encountered in the area. This is in contrast to previous studies assessing multiple hybrid zones in fishes (Aboim, Mavarez, Bernatchez, & Coelho, 2010; Nolte et al., 2009). These studies found differential patterns of introgression between two hybrid zones and attributed these different patterns to extrinsic factors that are differentially affecting the populations in different areas. In our study, shoal chub alleles were crossing more often into prairie chubs from both upstream and downstream reaches, and it is likely that at least some of these alleles are selectively advantageous regardless of where the individuals were spawned. It is unknown whether or not the hybrids analyzed in the upstream reaches actually spawned there or traveled from the downstream reaches; however, most of the hybrid individuals in the upstream reaches were late‐generation hybrids with largely prairie chub backgrounds.

### 4.2 Genetic and environmental associations

The distributions of both species and their hybrids in the Red River are strongly associated with several environmental variables, which are known to be important factors in structuring many other fish communities (Barlow, 1958; Ostrand, 2000; Ostrand & Wilde, 2001; 2004). Marie et al. (2012) found both positive and negative associations between physiochemical environmental conditions (e.g., temperature, dissolved oxygen, and pH) and hybridization rates, and suggested these may be limiting factors on fishes that are affecting their ability to reproduce in certain areas. Here, we found several water quality variables that are significant predictors of their genetic assignment probability (*q*), including specific conductance, pH, current velocity, depth, and dissolved oxygen. We found that temperature was not a significant predictor of *q*, which is in contrast to other published studies of fish hybridization (Marie et al., 2012; Yau & Taylor, 2013). In particular, specific conductance explained a majority of the variation in the model, indicating it was the strongest environmental predictor of the genomic composition of individuals that was measured, and as such may be a limiting factor in the distributions of these species. As specific conductance increases, one is more likely to encounter prairie chubs, whereas shoal chubs are more likely to be found in areas with lower specific conductance, and admixed individuals were captured more often in areas with intermediate specific conductance. This is an interesting association and future experimental studies specifically testing the overall fitness of prairie chubs, shoal chubs, and their hybrids at various water quality (e.g., specific conductance, pH, current velocity, and dissolved oxygen) levels are certainly warranted.

Prairie chubs are endemic to the upper Red River basin, which is classified as a prairie stream system having, on average, higher specific conductance and lower dissolved oxygen than the lower Red River (Higgins & Wilde, 2005; Ruppel et al., 2017). Additionally, there are no physical barriers preventing the prairie chub from moving lower in the basin toward Lake Texoma in larger numbers, and conversely there is no physical barrier preventing shoal chubs from moving upstream (except for the Wichita River). Considering the importance of water quality in shaping aquatic fish communities (Barlow, 1958; Marie et al., 2012; Ostrand, 2000; Ostrand & Wilde, 2001; 2004), this certainly presents an interesting hypothesis and warrants future experiments or studies to understand species restrictions.

## 5 CONCLUSIONS

Overall, we found an overall pattern of asymmetric hybridization, which could be due to the relative abundances of each species. In the zone of hybridization, shoal chubs are ~3x more abundant, thus providing more opportunities for reproduction with prairie chubs and hybrid individuals. There is also a broad pattern of asymmetric introgression, with shoal chub alleles tending to introgress into individuals comprised of predominately prairie chub genomic backgrounds. This asymmetric introgression may be due in large part to many of the shoal chub alleles being selectively advantageous. This could be concerning from a conservation standpoint with respect to the genetic integrity of the pure prairie chub populations. However, in other riverine fishes, pairs of species with two independent hybrid zones have had different asymmetries with regards to introgressing alleles, which has been attributed to extrinsic or localized environmental selection pressures (Aboim et al., 2010; Nolte et al., 2009), which is not the case here. We found that assignment probability was predicted by various water quality parameters (e.g. specific conducatnce, depth, dissolved oxygen, current velocity, and pH), indicating that the location of these species and their hybrids is highly associated with water quality. Thus, if the water quality was to change, potentially due to anthropogenic causes, which has been proposed in the past, such as attempting to decrease the salinity levels for agriculture use (U.S. Army Corps of Engineers, 2012), we may see shifts in the species distributions which could be detrimental for the imperiled prairie chub. Finally, this study not only confirms that the prairie chub is a distinct lineage, supporting the nominal taxonomy (Echelle et al., 2018; Eisenhour, 2004; Underwood et al., 2003), it is also the first study to reveal extensive introgression between the shoal chub and prairie chub in the Red River basin of Texas which is associated with various environmental variables, predominately specific conductance.

## AUTHORS' CONTRIBUTIONS

V.A.S., T.H.B., and N.H.M designed the research; V.A.S., T.H.B., and D.S.R. conducted the field work; V.A.S. conducted the laboratory work; V.A.S., C.C.N., and N.H.M. analyzed the data; V.A.S., D.S.R., C.C.N., T.H.B., and N.H.M. wrote the manuscript.

## Data Availability

Data (individual fastq files containing sequence reads, and environmental data from sampling sites) are currently accessible from the Dryad Digital Repository: https://doi.org/10.5061/dryad.dr06p8f.
