# Supplementary material for: Asymmetric introgression between fishes in the Red River basin of Texas is associated with variation in water quality
Source: Ecol Evol. 2019 Jan 24;9(4):2083–95. doi: 10.1002/ece3.4901 (PMC6392354; doi:10.1002/ece3.4901)
Supplement: Supplementary file 1 [file ECE3-9-2083-s001.docx]

Appendix Table 1. Confidence intervals (95%) of pairwise genetic differentiation (Nei’s G_ST_) between all sites where genetic analyses were performed. Lower bound confidence intervals are below the diagonal with upper bound confidence intervals above diagonal.

|  | BR_1462 | BR_290 | PR_104 | PR_6 | PR_283 | PR_287 | RR_89 | RR_81 | RR_35 | RR_79 | RR_70 | RR_283 | RR_259 | WR_1919 | WR_6 |
| --- | --- | --- | --- | --- | --- | --- | --- | --- | --- | --- | --- | --- | --- | --- | --- |
| BR_1462 | - | 0.004 | 0.395 | 0.387 | 0.380 | 0.387 | 0.232 | 0.194 | 0.232 | 0.296 | 0.320 | 0.391 | 0.192 | 0.387 | 0.390 |
| BR_290 | 0.004 | - | 0.389 | 0.380 | 0.374 | 0.381 | 0.226 | 0.189 | 0.226 | 0.290 | 0.314 | 0.384 | 0.186 | 0.381 | 0.383 |
| PR_104 | 0.387 | 0.381 | - | 0.015 | 0.017 | 0.017 | 0.077 | 0.112 | 0.099 | 0.035 | 0.024 | 0.025 | 0.169 | 0.013 | 0.015 |
| PR_6 | 0.378 | 0.372 | 0.015 | - | 0.009 | 0.008 | 0.069 | 0.105 | 0.091 | 0.027 | 0.016 | 0.017 | 0.161 | 0.005 | 0.007 |
| PR_283 | 0.372 | 0.366 | 0.017 | 0.009 | - | 0.010 | 0.065 | 0.099 | 0.086 | 0.026 | 0.017 | 0.019 | 0.154 | 0.007 | 0.009 |
| PR_287 | 0.379 | 0.373 | 0.016 | 0.008 | 0.010 | - | 0.070 | 0.105 | 0.092 | 0.028 | 0.017 | 0.018 | 0.161 | 0.006 | 0.008 |
| RR_89 | 0.227 | 0.221 | 0.074 | 0.067 | 0.063 | 0.067 | - | 0.010 | 0.010 | 0.022 | 0.048 | 0.074 | 0.030 | 0.069 | 0.072 |
| RR_81 | 0.190 | 0.185 | 0.108 | 0.101 | 0.096 | 0.101 | 0.010 | - | 0.015 | 0.044 | 0.075 | 0.109 | 0.020 | 0.105 | 0.107 |
| RR_35 | 0.227 | 0.221 | 0.095 | 0.088 | 0.083 | 0.088 | 0.009 | 0.014 | - | 0.036 | 0.067 | 0.095 | 0.030 | 0.091 | 0.094 |
| RR_79 | 0.290 | 0.284 | 0.034 | 0.026 | 0.025 | 0.027 | 0.021 | 0.042 | 0.035 | - | 0.020 | 0.034 | 0.083 | 0.026 | 0.029 |
| RR_70 | 0.314 | 0.307 | 0.023 | 0.016 | 0.016 | 0.017 | 0.046 | 0.072 | 0.064 | 0.020 | - | 0.025 | 0.123 | 0.015 | 0.017 |
| RR_283 | 0.383 | 0.376 | 0.024 | 0.016 | 0.018 | 0.018 | 0.071 | 0.105 | 0.092 | 0.033 | 0.024 | - | 0.164 | 0.015 | 0.017 |
| RR_259 | 0.186 | 0.181 | 0.163 | 0.155 | 0.149 | 0.156 | 0.029 | 0.019 | 0.029 | 0.080 | 0.119 | 0.159 | - | 0.162 | 0.165 |
| WR_1919 | 0.379 | 0.373 | 0.013 | 0.005 | 0.007 | 0.006 | 0.067 | 0.101 | 0.088 | 0.025 | 0.014 | 0.015 | 0.156 | - | 0.005 |
| WR_6 | 0.382 | 0.375 | 0.014 | 0.006 | 0.009 | 0.008 | 0.069 | 0.103 | 0.090 | 0.028 | 0.016 | 0.016 | 0.159 | 0.005 | - |
